# The application of square-stepping exercise in patients with stroke: a scoping review

**DOI:** 10.3389/fneur.2026.1952887

**Published:** 2026-10-12

**Authors:** Yali Yan, Juan Xu, Jiaxin Yao, Yuanyuan Liu, Mengdan Li, Xinxin Geng

**Affiliations:** 1 School of Nursing, Shanxi University of Chinese Medicine, Jinzhong, Shanxi, China; 2 Shanxi Provincial People’s Hospital, Taiyuan, Shanxi, China; 3 School of Nursing, Fujian University of Chinese Medicine, Fuzhou, Fujian, China

**Keywords:** cognitive function, motor function, scoping review, square-stepping exercise, stroke

## Abstract

**Background:**

Square-stepping exercise (SSE) is a form of dual-task training that has been used in studies involving patients with stroke. This scoping review provides an overview of the intervention protocols and outcomes reported in studies examining the use of SSE among patients with stroke.

**Methods:**

PubMed, Embase, Web of Science, The Cochrane Library, CINAHL, CNKI, SinoMed, Wanfang Data, and the VIP Database were searched for studies published from 2006, when the first study on SSE was published, to April 30, 2026. A total of 459 records were identified, of which seven met the eligibility criteria and were included in the final analysis.

**Results:**

A total of seven studies were included. The intervention types included face-to-face and hybrid interventions. The duration of a single session was generally 30 min, the frequency was mostly 3–5 times per week, and the intervention period ranged from 6 to 24 weeks. The outcome measures primarily covered two domains: motor function and cognitive function. The findings suggested that SSE may improve balance and gait function in patients with stroke and may reduce the risk of falls. In addition, SSE may improve global cognitive function, including executive function and attention.

**Conclusion:**

SSE may help improve motor and cognitive function in patients with stroke, but related research remains at an exploratory stage. Future research should include large-scale, multicenter studies with long-term follow-up to further clarify its long-term effects and optimal intervention protocol, explore remotely delivered SSE interventions, and promote the standardized implementation and dissemination of SSE in stroke rehabilitation.

## 1 Introduction

Stroke is an acute cerebrovascular disease and is currently the second leading cause of death and the third leading cause of disability-adjusted life years (DALYs) worldwide (1, 2). Studies have shown that 80% of patients experience limb motor dysfunction after stroke (3), while 50% experience varying degrees of cognitive impairment (4). Cognitive and motor dysfunctions often coexist, substantially affecting patients’ activities of daily living and rehabilitation progress while increasing their economic and social burden (5). Currently, rehabilitation approaches for post-stroke motor dysfunction mainly include various exercise therapies, mirror visual feedback, and suspension training (6). Interventions for cognitive impairment include pharmacological treatment, music therapy, electroacupuncture, and virtual reality technology (7). Although the aforementioned methods have achieved certain clinical benefits, they have primarily focused on improving a single functional domain. In addition, some of these interventions require specialized personnel, dedicated facilities, or sophisticated equipment, which may limit their accessibility and widespread implementation. Given the close interrelationship between motor and cognitive functions, improvements in motor function after stroke may be accompanied by improvements in cognitive function (8). Therefore, exploring convenient nonpharmacological rehabilitation approaches that can simultaneously improve both motor and cognitive functions is of considerable significance for promoting comprehensive functional recovery in patients with stroke.

Dual-task training (DTT) refers to the simultaneous or alternating performance of two or more motor and/or cognitive tasks (9), effectively simulating multitasking situations in daily life and demonstrating unique advantages in functional rehabilitation (10). Previous studies have demonstrated that DTT is superior to single-task training in improving gait performance and balance function and reducing the risk of falls in patients with stroke (11, 12). Multiple systematic reviews and meta-analyses have also shown that DTT provides comprehensive benefits for motor and cognitive functions in patients with stroke (13). However, DTT encompasses diverse training modalities, mainly including dual-task treadmill training (14), exergaming (15), and dance training (16). These modalities differ to some extent in terms of motor tasks, cognitive load, and training environment. For example, dual-task treadmill training primarily involves adding cognitive tasks during continuous walking, resulting in a relatively simple movement pattern; exergaming relies on visual, auditory, and interactive devices to provide external feedback and is highly interactive, but requires relatively sophisticated equipment and specialized training environments; dance training emphasizes rhythmic movements and complex movement combinations, placing relatively high demands on the motor abilities of patients with stroke.

In contrast, square-stepping exercise (SSE) is a form of dual-task training that combines visuospatial cognitive tasks with multidirectional stepping training and was first introduced by Shigematsu et al. in 2006 (17). Its core feature is the performance of stepping movements in forward, backward, lateral, and diagonal directions along predefined square paths. While improving lower-limb muscle strength and balance, SSE engages cognitive domains such as attention and executive function through a sequence of cognitive tasks involving step-sequence memorization, path planning, and error correction (18). Compared with traditional gait training, the unique value of SSE lies in its deep integration of motor execution and cognitive processing, thereby forming a closed-loop intervention encompassing physical activity, neural activation, and functional improvement (19). Furthermore, SSE requires no specialized equipment, can be conducted in a relatively small space, has relatively low implementation costs, and allows the training difficulty to be adjusted according to patients’ functional status, thereby facilitating its application in patients with different levels of functional ability after stroke (20).

To date, a scoping review has summarized the training protocols and outcome measures of SSE in older adults (21). However, older adults and patients with stroke differ in terms of functional impairments and rehabilitation needs, and the existing evidence cannot fully reflect the current application of SSE in stroke rehabilitation. In recent years, the application of SSE in stroke rehabilitation has gradually increased, demonstrating promising potential for improving balance ability, gait stability, and cognitive function in patients with stroke (22, 23). However, existing studies differ in terms of intervention format, exercise protocols, outcome measures, and treatment effects. In addition, variations in patient characteristics, functional status, and study designs have contributed to heterogeneity among study findings, hindering the clinical implementation of SSE in clinical nursing practice. Therefore, this study adopted a scoping review methodology to synthesize and analyze the literature on the application of square-stepping exercise in patients with stroke, with the aim of providing a reference for healthcare professionals in scientific research and clinical practice.

## 2 Materials and methods

This study strictly followed the scoping review framework proposed by Arksey and O’Malley (24), and the reporting adhered to the PRISMA-ScR guidelines (25).

### 2.1 Identifying the research questions

Based on a review of the relevant literature, the following research questions were formulated:

(1) What types of square-stepping exercise have been used in patients with stroke?
(2) How have the intervention protocols for square-stepping exercise been designed, including session duration, frequency, and intervention period?
(3) What outcome measures have been used to evaluate the implementation of square-stepping exercise, and what are its effects?

### 2.2 Inclusion and exclusion criteria

#### 2.2.1 Inclusion criteria

The inclusion criteria were established based on the PCC framework. Participants (P): patients with stroke who met the diagnostic criteria of the *Diagnostic Essentials for Major Cerebrovascular Diseases in China (2019)* (26) or other recognized diagnostic criteria, with the diagnosis confirmed by computed tomography (CT) or magnetic resonance imaging (MRI), regardless of the type or duration of hemiplegia; Concept (C): the application of square-stepping exercise in the rehabilitation of patients with stroke, including studies in which it was implemented independently or in combination with other rehabilitation interventions; Context (C): any setting in which patients are situated.

#### 2.2.2 Exclusion criteria

The exclusion criteria were as follows: (1) duplicate publications, studies with incomplete content, or studies for which the full text was unavailable; (2) research protocols, reviews, conference papers, dissertations, guidelines, and policy documents; and (3) studies published in languages other than Chinese or English.

### 2.3 Search strategy

A systematic search was conducted in PubMed, Web of Science, Embase, CINAHL, The Cochrane Library, CNKI, SinoMed, Wanfang Data, and the VIP Database to identify studies investigating the application of square-stepping exercise in stroke rehabilitation. The search covered the period from 2006, when the first study of SSE was published, through April 30, 2026. Both controlled vocabulary and free-text terms were used. The English-language search terms included “brain infarction,” “cerebral infarction,” “cerebral hemorrhage,” “ischemic stroke,” “apoplexy,” “cerebrovascular accident,” “stroke,” “square-stepping exercise,” “square step exercise”, “square steps”, “square step practice”. The PubMed search strategy is presented in Table 1.

**Table 1:** PubMed search strategy.

Step	Search strategy
#1	stroke[MeSH Terms] OR brain infarction[MeSH Terms] OR cerebral infarction[MeSH Terms] OR hemorrhagic stroke[MeSH] OR (ischemic stroke[MeSH Terms] OR cerebral hemorrhage[MeSH Terms]
#2	cerebrovascular accident[Title/Abstract] OR apoplexy[Title/Abstract] OR cerebral stroke[Title/Abstract] OR brain infarct[Title/Abstract] OR cerebrum hemorrhage[Title/Abstract] OR intracerebral hemorrhage[Title/Abstract] OR cerebral brain hemorrhage[Title/Abstract]
#3	#1 OR #2
#4	square-stepping exercise[Title/Abstract] OR square step exercise[Title/Abstract] OR square step practice[Title/Abstract] OR square steps[Title/Abstract]
#5	#3 AND #4

### 2.4 Literature screening

The retrieved bibliographic records were imported into EndNote X9 for deduplication. Two trained researchers independently screened the titles and abstracts according to the inclusion and exclusion criteria, followed by full-text screening to identify eligible studies. The two researchers also independently extracted data, including the first author, country, publication year, sample size, intervention format, intervention content, and outcome measures. Any disagreements during study selection or data extraction were resolved through discussion with a third researcher. In accordance with JBI guidance for scoping reviews (27) and the PRISMA-ScR statement (25), a quality appraisal of the included studies was not undertaken.

## 3 Results

### 3.1 Literature search results

The initial search yielded 459 literature records. After removing 152 duplicates, 307 records remained for title and abstract screening. Of these, 252 were excluded for not meeting the inclusion criteria, and 55 underwent full-text assessment. Following full-text assessment, a further 48 records were excluded: 11 had an ineligible study design, 17 involved ineligible interventions, 10 included an ineligible study population, 8 lacked extractable valid trial data, and 2 had unavailable full texts. Ultimately, 7 studies were included, comprising 3 articles published in English (28–30) and 4 articles published in Chinese (31–34). The detailed literature screening process is presented in Figure 1.

**Figure 1:**
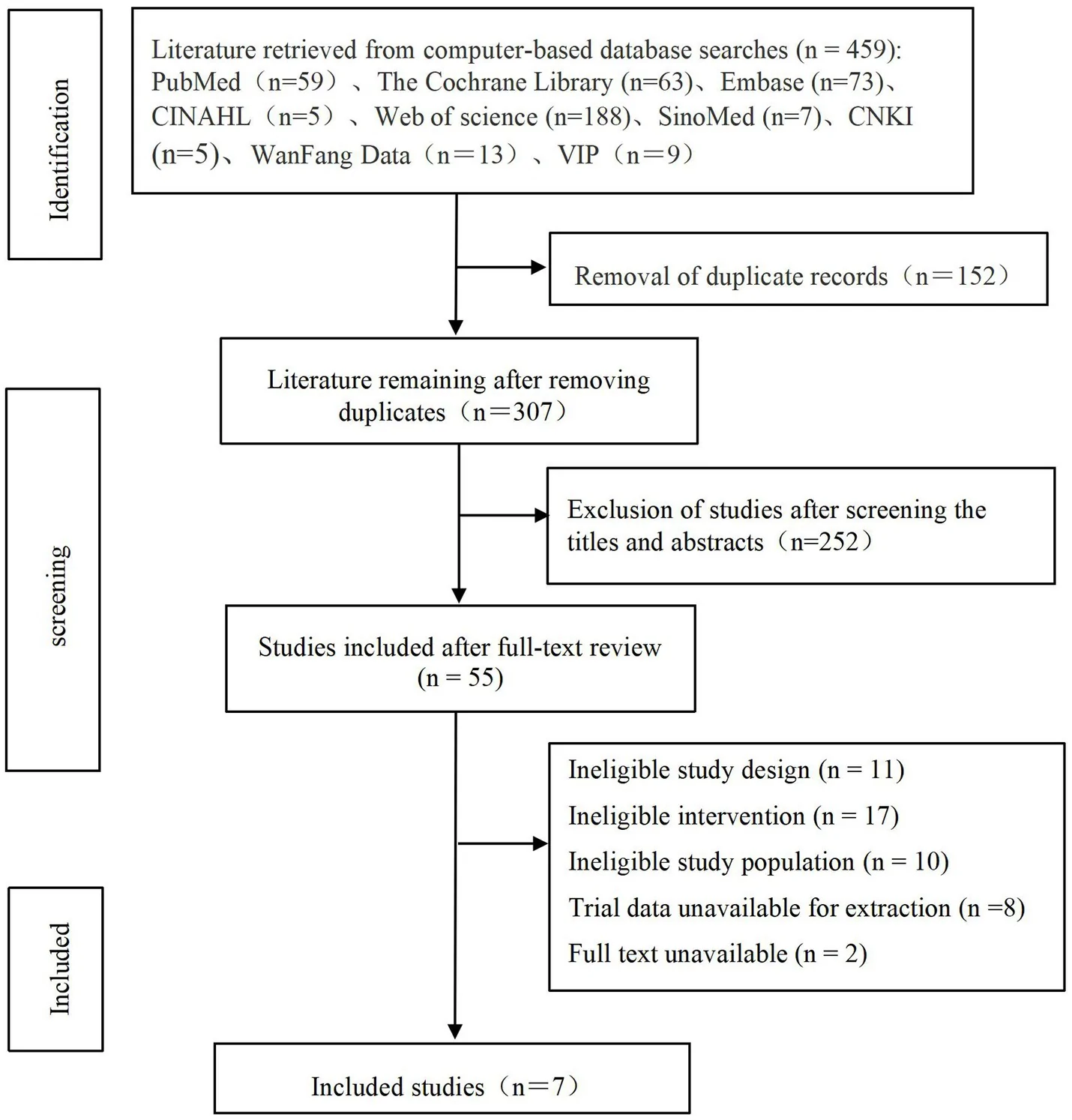
Flow diagram of literature screening.

### 3.2 Basic characteristics of the included studies

A total of seven studies were included. The included studies comprised five randomized controlled trials and two quasi-experimental studies. The studies were published between 2014 and 2026 and were conducted in China (*n* = 4), India (*n* = 2), and Brazil (*n* = 1). The total sample size was 357 participants. The characteristics of the included studies are presented in Tables 2, 3.

**Table 2:** Characteristics of included literature.

Author	Year	Location	Study design	Sample size (T/C)	Intervention type	Intervention group	Control group	Primary outcomes
Jiang et al. (31)	2025	China	RCT	30/30	Hybrid	3 or 4 times per week (+ 10 min warm-up), intervention period not reported	Routine rehabilitation nursing (including condition assessment, medication and rehabilitation guidance, health education, exercise and dietary guidance, and psychological support)	FMA, MoCA
Wang et al. (32)	2023	China	RCT	39/41	Hybrid	Once daily, 30 min/session (+10 min warm-up), for 24 weeks	Routine care (including health education, exercise rehabilitation, cognitive training, and post-discharge follow-up)	MoCA, BBS, MFS
Túbero et al. (28)	2014	Brazil	NRCT	*n* = 13	Face-to-face	3 times per week, 90 min/session, for 16 weeks	–	BBS, TUG, MMSE, BBRC
Zhang et al. (33)	2022	China	RCT	30/30	Face-to-face	5 times per week, 30 min/session (+10 min warm-up, 10 min cool-down stretching), for 12 weeks	Functional rehabilitation training for the affected limb (including dynamic and static balance, weight-bearing, gait, and body weight-shifting training)	Affected foot plantar surface area, sway length, mean pressure during walking
Yumlembam et al. (29)	2025	India	RCT	21/21	Face-to-face	5 times per week, 30 min/session, for 6 weeks	Routine physical therapy (including stretching, muscle strength, balance, gait, and core training)	BBS, TUG, FES-I
Chen et al. (34)	2025	China	RCT	41/41	Face-to-face	Once daily, 20 min/session, for 12 weeks	Routine care (including basic nursing care, medication and condition guidance, simple cognitive training, limb exercises, and psychological support)	MoCA, Barthel Index
Ahire et al. (30)	2026	India	NRCT	*n* = 20	Face-to-face	5 times per week, for 6 weeks, intervention duration not reported	–	RMI, MAS

T = intervention group; C = control group. Hybrid intervention: face-to-face + online. Outcome measures: FMA: Fugl-Meyer Assessment; MFS: Morse Fall Scale; BBS: Berg Balance Scale; TUG: Timed Up and Go test; RMI: Rivermead Mobility Index; MAS: Modified Ashworth Scale; MoCA: Montreal Cognitive Assessment; MMSE: Mini-Mental State Examination; BBRC: Brief Cognitive Screening Scale; FES-I: Falls Efficacy Scale-International; Barthel Index: Barthel Index for Activities of Daily Living.

**Table 3:** Main findings observed in the included studies.

Author	Summary of the findings
Jiang et al. (31)	SSE intervention effectively improves motor function and overall cognitive performance in patients with cognitive impairment following intracerebral hemorrhage.
Wang et al. (32)	SSE can improve cognitive function and balance ability in patients with post-stroke cognitive impairment and reduce the risk of falls.
Túbero et al. (28)	SSE improved dynamic balance and cognition in patients with cerebrovascular accident compared to physiotherapy group.
Zhang et al. (33)	SSE can improve balance and gait in patients with stroke, as evidenced by a shorter sway path length and increased mean pressure during walking in the intervention group after treatment.
Yumlembam et al. (29)	Compared with the control group, patients who received SSE intervention showed improved BBS scores, shorter TUG completion times, and a reduced risk of falls.
Chen et al. (34)	SSE can improve cognitive function and enhance the ability to perform activities of daily living in patients with post-stroke cognitive impairment.
Ahire et al. (30)	SSE can improve mobility and alleviate muscle spasticity in the affected limbs of patients with stroke.

### 3.3 Square-stepping exercise intervention content

#### 3.3.1 Training content

SSE uses a grid as the training platform and is performed on a dedicated training mat or on a marked floor surface. The training mat measures 250 cm in length and 100 cm in width and is divided into 40 square cells, each measuring 25 cm × 25 cm. The training includes more than 100 exercise patterns, with difficulty levels classified as beginner, intermediate, and advanced. Participants are required to complete the corresponding gait exercises according to instructions, and the movement patterns involve various complex combinations of stepping, including forward, backward, lateral, and diagonal movements (35, 36).

#### 3.3.2 Intervention types

The intervention formats used in the included studies were mainly of two types. (1) Face-to-face intervention: five studies adopted face-to-face intervention, in which professionals provided on-site instruction, supervision, and feedback in hospitals, rehabilitation centers, and other settings (28–30, 33, 34). (2) Hybrid intervention (face-to-face + online): two studies adopted a hybrid intervention model combining face-to-face instruction with online remote follow-up. That is, professional personnel provided training instruction in the hospital, while home-based rehabilitation training was followed up outside the hospital through methods such as WeChat check-ins, and the patients’ mastery of the training content was assessed (31, 32).

#### 3.3.3 Intervention duration, frequency, and period

Among the seven included studies, the duration, frequency, and period of the exercise intervention varied according to the intervention goals and the participants’ individual conditions. (1) Intervention duration: three studies used a duration of 30 min (29, 32, 33), one study used 20 min (34), and one study used 90 min (28). (2) Intervention frequency: three studies required exercise 5 times per week (29, 30, 33), and two studies required exercise 3–4 times per week (28, 31). (3) Intervention period: the intervention period varied considerably, ranging from 6 weeks (29) to as long as 6 months (32).

#### 3.3.4 Outcome measures and intervention effects

The outcome measures used in the included studies mainly covered motor function, cognitive function, and activities of daily living (ADL). (1) Motor function: The Fugl-Meyer Assessment (FMA) and the Rivermead Mobility Index (RMI) were used to evaluate patients’ functional recovery. Balance was assessed using the Berg Balance Scale (BBS) and the Timed Up and Go test (TUG). Fall risk was assessed using the Morse Fall Scale (MFS) and the Falls Efficacy Scale–International (FES-I). The Modified Ashworth Scale (MAS) was used to measure muscle tone. In addition, one study (33) used objective indicators, such as the plantar surface area of the affected side, sway-path length, and mean plantar pressure during walking, to evaluate postural control and gait characteristics. (2) Cognitive function: Cognitive function was mainly assessed using the Montreal Cognitive Assessment (MoCA), the Mini-Mental State Examination (MMSE), and the Brief Cognitive Screening Test (BBRC). (3) Activities of daily living: The Barthel Index was used to assess ADL.

Four studies showed that SSE improved balance in patients with stroke (28, 29, 32, 33). Two studies reported that SSE reduced fall risk (29, 32). One study found that SSE improved gait characteristics, as evidenced by a reduction in gait variability (33). Another study focused on muscle tone and showed that SSE reduced muscle tone (30). In addition, four studies indicated that SSE enhanced cognitive function in patients with stroke, particularly in the domains of attention, orientation, and memory (28, 31, 32, 34). Furthermore, one study reported that SSE improved activities of daily living (ADL) in patients with stroke (34).

## 4 Discussion

### 4.1 Face-to-face intervention is the main mode of SSE implementation, but the application prospects of remote SSE are worthy of attention

In stroke rehabilitation practice, SSE is still primarily delivered through face-to-face interventions. This approach helps bridge the gap between instructors and patients, enabling the timely identification and correction of movement deviations during training and thereby ensuring training quality. However, it is constrained by the schedules of both parties and travel costs, making it difficult to meet patients’ long-term home-based rehabilitation needs. Therefore, Jiang et al. (31) and Wang et al. (32) explored a hybrid intervention model combining in-hospital face-to-face instruction with out-of-hospital remote supervision. Relying on remote communication technologies, this model provides training guidance, interactive question-and-answer support, and feedback after discharge. It offers advantages such as real-time communication, convenience, rapidity, and low cost, which may, to some extent, compensate for the temporal and spatial limitations of traditional face-to-face interventions (37, 38). However, the implementation modalities of remote SSE in the two studies were relatively limited (31, 32), relying primarily on training check-ins, text-based communication, or video feedback. These approaches make it difficult to continuously monitor training quality and movement performance and to fully meet patients’ individualized rehabilitation needs. Nevertheless, the use of mobile applications provides practical implications for remote interventions in patients with stroke. Shellington et al. (39) showed that the HealtheBrain app could effectively deliver remote SSE training to older adults while accommodating individual differences and supporting progressive training through features such as personalized step-length calibration, real-time movement assessment, staged task setting, and a virtual reward system. The study confirmed the feasibility of this approach and demonstrated its potential to improve cognitive function. However, the app is primarily intended for older adults and was developed by an overseas team. It therefore has certain limitations in terms of language adaptation, technical compatibility, and disease specificity, making it difficult to apply directly to patients with stroke in China. Future research could therefore integrate the rehabilitation characteristics of patients with stroke to develop appropriate mobile support tools, so as to improve the accessibility and continuity of remote SSE training.

### 4.2 The application of SSE in patients with stroke has shown positive effects, but no consensus has yet been reached on the intervention protocol

Six studies (28–33) showed that SSE improved lower-limb function and balance in patients with stroke and may help reduce the risk of falls. Through multidirectional stepping patterns, including forward, backward, lateral, and turning steps, SSE purposefully trains patients to actively control their center of mass and maintain stability in multiple planes during movement. Compared with conventional walking, SSE requires patients to integrate vestibular, proprioceptive, and visual inputs while continuously disrupting and restoring postural equilibrium. These inputs are rapidly processed by the cerebellum and basal ganglia to generate precise postural adjustments, thereby enabling patients to respond to complex postural changes (40). Post-stroke sequelae often lead to impaired postural responses in directions such as lateral and backward, which is a key factor contributing to an increased risk of falls (41). Studies have shown that traditional straight-line walking may be insufficient to address these deficits, whereas SSE, through structured and repetitive multidirectional challenges, enhances weight shifting and postural strategies, thereby effectively improving gait stability and reducing the risk of falls (42). Furthermore, Zhang et al. (33) found that following systematic SSE intervention, gait variability in patients with stroke improved, as indicated by a shorter sway length and increased mean pressure during walking. These findings suggest that SSE may promote adjustments in lower-limb movement patterns by improving weight-bearing capacity on the affected side and plantar pressure distribution, thereby reducing bilateral weight-bearing asymmetry during walking and optimizing gait characteristics. In addition to motor function, four studies (28, 31, 32, 34) demonstrated that SSE improved global cognitive function in patients with stroke. The decline in post-stroke cognitive function may be associated with reduced levels of brain-derived neurotrophic factor (BDNF) and insulin-like growth factor-1 (IGF-1) in specific brain regions (43). A study showed that SSE stimulates brain cells by requiring patients to perform memory tasks involving step patterns, thereby increasing blood BDNF levels, maintaining IGF-1 levels, promoting neurogenesis and angiogenesis, and exerting positive effects on neural function, consequently improving cognitive function (44). Furthermore, during SSE training, step patterns with progressively increasing memory demands constitute a visuospatial working memory task that can continuously reinforce memory training and increase task difficulty, thereby improving working memory ability and, consequently, cognitive function in patients with stroke (45). Compared with conventional dual-task training, the cognitive task in SSE serves as a prerequisite for the motor task and may simultaneously produce motor and cognitive benefits, thereby exerting a synergistic effect on brain plasticity (46).

Although SSE has shown positive effects in stroke rehabilitation, no standardized intervention protocol has yet been established. In the included studies, the intervention frequency was mostly 3–5 times per week (28–33), and the duration of each session varied considerably, ranging from 20 to 90 min. These differences may be related to variations in study objectives, environmental conditions, or participant characteristics. Currently, insufficient evidence is available to determine whether extending the duration of each session or increasing training frequency can further improve rehabilitation outcomes. Therefore, SSE intervention protocols should be patient-centered and should fully consider patients’ acceptance. The duration of each intervention should follow the principle of gradual progression, increasing gradually and being flexibly adjusted according to the patient’s physical condition, tolerance of cognitive load, and subjective willingness. Treatment sessions that are too short should be avoided to prevent inadequate rehabilitation effects, while excessively prolonged sessions should also be avoided to prevent patient fatigue, thereby ensuring the safety and efficacy of the intervention. In addition, stroke rehabilitation is a long-term and dynamically evolving process (47), whereas the follow-up periods in the included studies were generally short (≤6 months), limiting the assessment of the long-term effects of SSE. For example, Wang et al. found that the risk of falls did not decrease significantly after 1 month of SSE intervention but was significantly lower in the intervention group than in the control group after three and 6 months (32). Therefore, future studies should conduct large-sample, multicenter studies with long-term follow-up to further compare the efficacy of different training frequencies, session durations, and intervention periods, and to determine the optimal intervention dose and dose–response relationship of SSE. This will provide more robust evidence-based support for developing standardized and individualized SSE intervention protocols and facilitating their clinical implementation.

### 4.3 Application prospects and practical implications of SSE in patients with stroke

As global healthcare increasingly emphasizes reducing reliance on pharmacological treatments, modern healthcare systems are gradually shifting toward nonpharmacological intervention models. SSE, as a safe and low-cost dual-task training approach, is characterized by a structured, quantifiable, and standardized training protocol. It also supports progressive training by adjusting stepping difficulty to meet the needs of patients with stroke at different stages of rehabilitation (19). Although the application of SSE in stroke rehabilitation remains exploratory, it aligns with the modern medical concept of “active rehabilitation” and has considerable potential for clinical dissemination. However, current evidence remains limited, and future research should focus on the following aspects.

(1) Safety management during SSE implementation should be strengthened. The included studies only stated that training should follow the principle of “from easy to difficult and from simple to complex,” but they lacked safety monitoring during the training process. Therefore, future studies should, based on an assessment of patients’ physical condition, cognitive-load tolerance, and subjective willingness, dynamically adjust the complexity, pace, and intensity of training by using subjective assessments, such as the Borg Rating of Perceived Exertion scale, together with objective measures, including blood pressure and heart rate, to avoid excessive fatigue (48, 49). At the same time, patients should be closely monitored for discomfort such as dizziness, chest pain, palpitations, and joint pain and instructed to adhere to the principle of “stopping immediately if pain or discomfort occurs” to ensure training safety (50). (2) Cultivate professional talent. The implementation of SSE requires instructors to possess comprehensive competencies, including accurate assessment, movement demonstration, intensity regulation, risk identification, and emergency response. However, there is currently a severe shortage of interdisciplinary professionals with knowledge of specialized nursing, exercise rehabilitation, and cognitive training, which has become an obstacle to the high-quality promotion of SSE. Therefore, future efforts could integrate square-stepping exercise into medical curricula and provide specialized certification training for relevant professionals to cultivate square-stepping exercise instructors (47). (3) Individualized exercise programs should be developed. The included studies involved diverse populations, such as patients with post-stroke cognitive impairment (31, 32, 34), patients with hemiplegia (30), and patients with sequelae of cerebrovascular accident (28). Because the functional status of different study populations varies, a single fixed protocol is unlikely to meet the needs of precision rehabilitation. Future studies could use standardized assessment tools to perform multidimensional stratification of patients with stroke and develop differentiated interventions for groups with different levels of motor and cognitive function and baseline comorbidity burden.

### 4.4 Limitations

(1) Heterogeneity in study design and study populations. Some of the included studies had small sample sizes and lacked randomized designs, which may have resulted in insufficient statistical power, selection bias, and confounding, thereby affecting the stability of the findings to some extent. In addition, the study populations exhibited a degree of heterogeneity, including patients with post-stroke cognitive impairment, intracerebral hemorrhage accompanied by cognitive impairment, and hemiplegia, which may increase the complexity of interpreting the findings. This study also did not assess study quality, which may have had a potential impact on the conclusions.
(2) Heterogeneity in outcome measures. The outcome measures used in the current studies varied, covering multiple domains, including balance function, lower-limb motor function, fall risk, and cognitive function. Although this diversity helps reflect the potential effects of SSE on functional recovery in patients with stroke from different perspectives, it increases the difficulty of comparing results across studies and synthesizing the evidence. Future studies should further standardize outcome measures and explore the development of a core outcome set (COS) to improve the comparability of research findings.
(3) Literature search and publication bias. The literature search was limited to Chinese- and English-language databases, and evidence from non-Chinese- or non-English-speaking regions may have been overlooked, potentially affecting the external validity of the findings due to regional differences. In addition, this study was based primarily on published literature; unpublished studies or studies not indexed in the databases may not have been included. Therefore, the potential influence of publication bias cannot be excluded.
(4) Limitations of the available evidence. The conclusions of the included studies tended to indicate that SSE may be beneficial, which may be related to the small number of included studies, the diversity of outcome measures, and the limited methodological quality of some studies. Therefore, the stability and certainty of the existing evidence remain subject to certain limitations. In addition, no studies have directly compared SSE with more established motor-cognitive training approaches, such as tai chi and yoga. The advantages of SSE over other forms of dual-task training remain to be further verified.

## 5 Conclusion

This scoping review examined the application of SSE in stroke rehabilitation and summarized the intervention content, delivery modalities, protocols, and outcomes reported in the studies. The findings suggest that SSE may have potential value in stroke rehabilitation and may contribute to improvements in motor and cognitive function among patients with stroke. However, no unified standards have yet been established regarding the duration, frequency, or overall intervention period of SSE. In addition, remote SSE has primarily relied on exercise check-ins, text-based communication, and video feedback, whereas continuous monitoring of training quality and performance, as well as individualized guidance, remains limited. Future studies should develop scientifically rigorous and standardized SSE intervention protocols, actively explore the application of information technologies, such as mobile applications, in remote rehabilitation, and further evaluate the effects of SSE through large-scale, multicenter studies with long-term follow-up. These efforts may facilitate the integration of SSE into stroke rehabilitation practice and potentially contribute to improved health outcomes and quality of life.

## Data Availability

The original contributions presented in the study are included in the article/Supplementary material, further inquiries can be directed to the corresponding author.
