# Supplementary material for: The application of square-stepping exercise in patients with stroke: a scoping review
Source: Front Neurol. 2026 Oct 12;17:1952887. doi: 10.3389/fneur.2026.1952887 (PMC13696318; doi:10.3389/fneur.2026.1952887)
Supplement: Supplementary file 1 [file Table_1.DOCX]

Supplementary Material

****1. Search results****

**Search cutoff date: April 2026**

**Search terms:**

卒中/脑卒中/脑出血/脑梗死/脑血管疾病/脑缺血/脑栓塞/中风/脑血管意外

方步运动/方块踏步运动

stroke/brain infarction/cerebral infarction/hemorrhagic stroke/ischemic stroke/cerebral hemorrhage/cerebrovascular accident/apoplexy/cerebral stroke/brain infarct/cerebrum hemorrhage/intracerebral hemorrhage/cerebral brain hemorrhage

square-stepping exercise/square step exercise/square step practice/square steps

CNKI：5

(SU %= '卒中' OR SU %= '脑卒中' OR SU %= '脑出血' OR SU %= '脑梗死' OR SU %= '脑血管疾病' OR SU %= '脑缺血' OR SU %= '脑栓塞' OR SU %= '中风' OR SU %= '脑血管意外') AND (SU %= '方步运动' OR SU %= '方块踏步运动')

PubMed:59

| #1 | stroke[MeSH Terms] OR brain infarction[MeSH Terms] OR cerebral infarction[MeSH Terms] OR hemorrhagic stroke[MeSH] OR （ischemic stroke[MeSH Terms] OR cerebral hemorrhage[MeSH Terms] |
| --- | --- |
| #2 | cerebrovascular accident[Title/Abstract] OR apoplexy[Title/Abstract] OR cerebral stroke[Title/Abstract] OR brain infarct[Title/Abstract] OR cerebrum hemorrhage[Title/Abstract] OR intracerebral hemorrhage[Title/Abstract] OR cerebral brain hemorrhage[Title/Abstract] |
| #3 | #1 OR #2 |
| #4 | square-stepping exercise[Title/Abstract] OR square step exercise[Title/Abstract] OR square step practice[Title/Abstract] OR square steps[Title/Abstract] |
| #5 | #3 AND #4 |

Web of science:188

| #1 | Stroke (Topic) OR brain infarction (Topic) OR cerebral infarction (Topic) OR hemorrhagic stroke (Topic) OR ischemic stroke (Topic) OR cerebral hemorrhage (Topic) OR cerebrovascular accident (Topic) OR apoplexy (Topic) OR cerebral stroke (Topic) OR brain infarct (Topic) OR cerebrum hemorrhage (Topic) OR intracerebral hemorrhage (Topic) OR cerebral brain hemorrhage (Topic) |
| --- | --- |
| #2 | square-stepping exercise (Topic) OR square step exercise (Topic) OR square step practice (Topic) OR square steps (Topic) |
| #3 | #1 AND #2 |

Embase:73

| #1 | 'cerebrovascular accident'/exp |
| --- | --- |
| #2 | 'accident, cerebrovascular':ti,ab,kw OR 'acute cerebrovascular lesion':ti,ab,kw OR 'acute focal cerebral vasculopathy':ti,ab,kw OR 'acute stroke':ti,ab,kw OR 'apoplectic stroke':ti,ab,kw OR 'apoplexia':ti,ab,kw OR 'apoplexy':ti,ab,kw OR 'blood flow disturbance, brain':ti,ab,kw OR 'brain accident':ti,ab,kw OR 'brain attack':ti,ab,kw OR 'brain blood flow disturbance':ti,ab,kw OR 'brain insult':ti,ab,kw OR 'brain insultus':ti,ab,kw OR 'brain vascular accident':ti,ab,kw OR 'cerebral apoplexia':ti,ab,kw OR 'cerebral insult':ti,ab,kw OR 'cerebral stroke':ti,ab,kw OR 'cerebral vascular accident':ti,ab,kw OR 'cerebral vascular insufficiency':ti,ab,kw OR 'cerebro vascular accident':ti,ab,kw OR 'cerebrovascular arrest':ti,ab,kw OR 'cerebrovascular failure':ti,ab,kw OR 'cerebrovascular injury':ti,ab,kw OR 'cerebrovascular insufficiency':ti,ab,kw OR 'cerebrovascular insult':ti,ab,kw OR 'cerebrum vascular accident':ti,ab,kw OR 'cryptogenic stroke':ti,ab,kw OR 'cva':ti,ab,kw OR 'insultus cerebralis':ti,ab,kw OR 'ischaemic seizure':ti,ab,kw OR 'ischemic seizure':ti,ab,kw OR 'stroke':ti,ab,kw OR 'thrombotic stroke':ti,ab,kw OR 'cerebrovascular accident':ti,ab,kw |
| #3 | #1 OR #2 |
| #4 | 'square-stepping exercise':ti,ab,kw OR 'square step exercise':ti,ab,kw OR 'square step practice':ti,ab,kw OR 'square steps':ti,ab,kw OR sse:ti,ab,kw |
| #5 | #3 AND #4 |
| #6 | application:ti,ab,kw OR rehabilitation:ti,ab,kw OR recovery:ti,ab,kw OR 'rehabilitation therapy':ti,ab,kw OR balance:ti,ab,kw OR gait:ti,ab,kw OR cognition:ti,ab,kw OR falls:ti,ab,kw OR function:ti,ab,kw |
| #7 | #6 AND #5 |

The Cochrane Library:63

CINAHL:2

| #1 | SU stroke OR TI (stroke OR “brain infarction” OR “cerebral infarction” OR “hemorrhagic stroke” OR “ischemic stroke” OR “cerebral hemorrhage” OR “cerebrovascular accident” OR “apoplexy” OR “cerebral stroke” OR “brain infarct” OR “cerebrum hemorrhage” OR “intracerebral hemorrhage” OR “cerebral brain hemorrhage”) OR AB (stroke OR “brain infarction” OR “cerebral infarction” OR “hemorrhagic stroke” OR “ischemic stroke” OR “cerebral hemorrhage” OR “cerebrovascular accident” OR “apoplexy” OR “cerebral stroke” OR “brain infarct” OR “cerebrum hemorrhage” OR “intracerebral hemorrhage” OR “cerebral brain hemorrhage”) |
| --- | --- |
| #2 | TI (“square-stepping exercise” OR “square step exercise” OR “square step practice” OR “square steps”) OR AB (“square-stepping exercise” OR “square step exercise” OR “square step practice” OR “square steps”) |
| #3 | #1 AND #2 |

**2. Reasons for exclusion of ineligible literature**

| Title | Reasons for exclusion of ineligible literature |
| --- | --- |
| The Test Retest Reliability and Convergent Validity of the Six Spot Step Test in Subacute Stroke Patients | Mismatch in interventions：Six Spot Step Test |
| The Four Square Step Test is a feasible and valid clinical test of dynamic standing balance for use in ambulant people poststroke | Mismatch in interventions：Four Square Step Test |
| The Reliability and Validity of the Four Square Step Testin Stroke Patients | Mismatch in interventions：Four Square Step Test |
| Evaluation of the Utility of the Modified Four Square Step Test in Subacute Stroke Patient | Mismatch in interventions：Four Square Step Test |
| Reliability and concurrent validity of Four Square Step Test scores in subjects with chronic stroke: a pilot study | Mismatch in interventions：Four Square Step Test |
| Early multidimensional mobility assessments for discriminating discharge-level ambulation and functional independence in subacute stroke inpatients:a retrospective cohort study | Mismatch in interventions |
| Reliability and validity of the single- and dual-task Figure-of-8 Walk tests in stroke patients | Mismatch in interventions and study types |
| A Study of the Clinical Utility of the Four Square Step Test for Predicting Falls in Stroke Patients | Mismatch in interventions：Four Square Step Test |
| The validity and reliability of the four square step test in different adult populations: a systematic review | Review |
| Development of the Modified Four Square Step Test and its reliability and validity in people with stroke | Mismatch in interventions：Four Square Step Test |
| Stepping towards health: a scoping review of square-stepping exercise protocols and outcomes in older adults | Review |
| Effects of square stepping exercise in patients with sequel of cerebrovascular accident | Include |
| Effective Combined Assessments of Weight Bearing Ratio and Four Square Step Test in Predicting Falls in Discharged Stroke Patients | Mismatch in interventions：Four Square Step Test |
| The effect of different exercise training types on functionality in older fallers a pilot randomized controlled trial. | Mismatch in study population |
| 智力PASS模型理论引导的方步运动联合言语听觉反馈在脑卒中认知障碍患者护理中的应用 | Include |
| Concurrent Validity between Figure-of-8 Walking Test and Functional Tests Included Tasks for Dynamic Balance and Walking in Patient with Stroke | Mismatch in interventions |
| 方步运动在脑卒中后认知障碍病人康复中的应用研究进展 | Include |
| The healthy mind, healthy mobility trial: a novel exercise program for older adults. | Mismatch in study population |
| Square-stepping Exercise Program effects on fall-related fitness and BDNF levels in older adults in Korea: a Randomized  Controlled Trial | Mismatch in study population |
| 言语听觉反馈认知训练联合PASS理论方步运动方案在脑出血认知障碍患者中的应用 | Include |
| Effects of Cognitive Versus mind-motor training on Cognition and Functional skills in the CommunityDwelling older adults. | Mismatch in study population |
| Home-based, square-stepping exercise program among older adults with multiple sclerosis: results of a feasibility randomized controlled study | Mismatch in study population |
| 基于PASS理论的方步运动方案在卒中后认知障碍患者中的应用 | Include |
| 方块踏步运动对脑卒中患者平衡及步态的影响 | Include |
| Does square-stepping exercise improve balance in unilateral middle cerebral artery stroke survivors? | Duplicate of included literature with only minor differences in title |
| Effect of a square-stepping exercise program on functional mobility in hemiparetic individuals | Full‑text not available |
| 方步运动的概念分析 | Review |
| 方步运动在老年人运动认知风险综合征预防中的应用进展 | Review |
| 方步运动在预防老年人跌倒中的应用进展 | Review |
| 四方步运动在脑卒中患者康复护理中的应用研究 | Mismatch in interventions |
| Effects of “Square Stepping Exercise” on motor and cognitive skills in Autism Spectrum Disorders children and adolescents | Mismatch in study population |
| Improving Balance and Lower Limb Coordination After Stroke Using Square Stepping Exercise | Trial registration |
| Memory function and Brain Functional Connectivity adaptations followingmultiple-modality Exercise and mind–motor training in older adults at risk of dementia: an exploratory Sub-study | Mismatch in interventions and study population |
| Does square-stepping exercise improve balance and walking speed in people recovering from stroke? | Trial registration |
| Does square-stepping exercise improve balance in unilateral middle cerebral artery stroke survivors? A randomized controlled clinical trial | Include |
| Effect of Square Stepping Exercise on Gait Performance in Post Stroke | Trial registration |
| Effects of square stepping exercise in patients with sequelae of stroke | Duplicate of included literature with only minor differences in title |
| Study on the influence of square step exercise on balance and cognitive function in stroke patients and its neural mechanism | Trial registration |
| A novel exercise for improving lower-extremity functional fitness in the elderly | Mismatch in study population |
| [Effects of cognitive and motor dual task training on gait performance in individuals with stroke-a pilot study](https://www.cochranelibrary.com/central/doi/10.1002/central/CN-01126454/full) | Mismatch in interventions: motor-cognitive dual-task training without SSE training modality |
| [The Feasibility of non-motorized Treadmill Training on Lower Extremity Muscle Strength, Balance, and Gait in Patients with Stroke: a Pilot Study](https://www.cochranelibrary.com/central/doi/10.1002/central/CN-02846851/full) | Mismatch in interventions: non-motorized treadmill training |
| 方块踏步练习对老年人身体下肢体适能的影响研究 | Conference abstract |
| [Time Pressure Motor Training With Step Square Exercise on Stroke Patients](https://www.cochranelibrary.com/central/doi/10.1002/central/CN-02866994/full) | Full‑text not available |
| [Intervention effect of Interactive Cognitive Motor Training on stroke patients](https://www.cochranelibrary.com/central/doi/10.1002/central/CN-02606852/full) | Mismatch in interventions: motor-cognitive dual-task training without SSE training modality |
| Effects of Square‐Stepping Exercise Training on the Attention, Dynamic Balance and Lower Extremity Motor Coordination. | Mismatch in study population |
| Comparative analysis of square stepping exercise and Swiss ball exercise for enhancing balance and preventing falls in older adults: A cross-sectional study. | Mismatch in study types: cross-sectional study |
| Impacts of Square Stepping Exercise on Physical-Cognitive Function, Biomarkers, Body Composition and Mental Health in Healthy Senior Aged 60 and Above: A Systematic Review. | Review |
| [Role of Stepping and BOSU Ball Training in Enhancing Postural Balance and Performance of Badminton Players](https://www.cochranelibrary.com/central/doi/10.1002/central/CN-02966945/full) | Trial registration |
| [Improving Balance and Lower Limb Coordination After Stroke Using Square Stepping Exercise](https://www.cochranelibrary.com/central/doi/10.1002/central/CN-03043229/full) | Trial registration |
| [Improving balance with wobble board exercises in stroke patients: single-blind, randomized clinical trial](https://www.cochranelibrary.com/central/doi/10.1002/central/CN-01980266/full) | 1.1.1 Mismatch in interventions: balance board training |
| [Investigating the Effects of Dual-Tasking with Virtual Reality on Stroke Patients](https://www.cochranelibrary.com/central/doi/10.1002/central/CN-02887544/full) | Mismatch in interventions |
| The effects of visual information deprivation and feedback balance training on balance in patients with stroke | Mismatch in interventions |
| 基于PASS理论的联合治疗对帕金森患者的干预效果 | Mismatch in study population |
| 不同运动方式对老年人跌倒效能影响的网状Meta分析 | Review |
| 方块踏步运动在老年人群中应用的研究进展 | Review |
| 方块踏步运动对老年人认知能力的影响综述 | Conference abstract |

**3.Preferred Reporting Items for Systematic reviews and Meta-Analyses extension for Scoping Reviews (PRISMA-ScR) Checklist**

| **SECTION** | **ITEM** | **PRISMA-ScR CHECKLIST ITEM** | **REPORTED ON PAGE #** |
| --- | --- | --- | --- |
| **TITLE** | | | |
| Title | 1 | Identify the report as a scoping review. | 1-2 |
| **ABSTRACT** | | | |
| Structured summary | 2 | Provide a structured summary that includes (as applicable): background, objectives, eligibility criteria, sources of evidence, charting methods, results, and conclusions that relate to the review questions and objectives. | 11-29 |
| **INTRODUCTION** | | | |
| Rationale | 3 | Describe the rationale for the review in the context of what is already known. Explain why the review questions/objectives lend themselves to a scoping review approach. | 30-85 |
| Objectives | 4 | Provide an explicit statement of the questions and objectives being addressed with reference to their key elements (e.g., population or participants, concepts, and context) or other relevant key elements used to conceptualize the review questions and/or objectives. | 89-95 |
| **METHODS** | | | |
| Protocol and registration | 5 | Indicate whether a review protocol exists; state if and where it can be accessed (e.g., a Web address); and if available, provide registration information, including the registration number. | Click here to enter text. |
| Eligibility criteria | 6 | Specify characteristics of the sources of evidence used as eligibility criteria (e.g., years considered, language, and publication status), and provide a rationale. | 96-110 |
| Information sources* | 7 | Describe all information sources in the search (e.g., databases with dates of coverage and contact with authors to identify additional sources), as well as the date the most recent search was executed. | 111-119 |
| Search | 8 | Present the full electronic search strategy for at least 1 database, including any limits used, such that it could be repeated. | 517-526 |
| Selection of sources of evidence† | 9 | State the process for selecting sources of evidence (i.e., screening and eligibility) included in the scoping review. | 120-128 |
| Data charting process‡ | 10 | Describe the methods of charting data from the included sources of evidence (e.g., calibrated forms or forms that have been tested by the team before their use, and whether data charting was done independently or in duplicate) and any processes for obtaining and confirming data from investigators. | 120-128 |
| Data items | 11 | List and define all variables for which data were sought and any assumptions and simplifications made. | 120-128 |
| Critical appraisal of individual sources of evidence§ | 12 | If done, provide a rationale for conducting a critical appraisal of included sources of evidence; describe the methods used and how this information was used in any data synthesis (if appropriate). | 120-128 |
| Synthesis of results | 13 | Describe the methods of handling and summarizing the data that were charted. | 120-128 |
| **RESULTS** | | | |
| Selection of sources of evidence | 14 | Give numbers of sources of evidence screened, assessed for eligibility, and included in the review, with reasons for exclusions at each stage, ideally using a flow diagram. | 131-137 |
| Characteristics of sources of evidence | 15 | For each source of evidence, present characteristics for which data were charted and provide the citations. | 139-142 |
| Critical appraisal within sources of evidence | 16 | If done, present data on critical appraisal of included sources of evidence (see item 12). | 534 |
| Results of individual sources of evidence | 17 | For each included source of evidence, present the relevant data that were charted that relate to the review questions and objectives. | 144-187 |
| Synthesis of results | 18 | Summarize and/or present the charting results as they relate to the review questions and objectives. | 536 |
| **DISCUSSION** | | | |
| Summary of evidence | 19 | Summarize the main results (including an overview of concepts, themes, and types of evidence available), link to the review questions and objectives, and consider the relevance to key groups. | 189-301 |
| Limitations | 20 | Discuss the limitations of the scoping review process. | 302-328 |
| Conclusions | 21 | Provide a general interpretation of the results with respect to the review questions and objectives, as well as potential implications and/or next steps. | 330-340 |
| **FUNDING** | | | |
| Funding | 22 | Describe sources of funding for the included sources of evidence, as well as sources of funding for the scoping review. Describe the role of the funders of the scoping review. | 451-354 |
